# Assessment of precision medicine potential in diabetes mellitus: A meta‐regression analysis of dose‐dependent glycaemic control data from 44 randomised controlled trials

**DOI:** 10.1111/dom.70066

**Published:** 2025-08-27

**Authors:** Kris G. Vargas, Benedikt Siemes, Tobias Rütten, Maximilian Brockmeyer, Kurt Huber, Georg Wolff, Oliver Kuss

**Affiliations:** ^1^ Clinic of Cardiology Klinikum Ibbenbüren Ibbenbüren Germany; ^2^ Division of Cardiology, Pulmonology and Vascular Medicine, Department of Internal Medicine Medical Faculty and University Hospital Düsseldorf, Heinrich Heine University Düsseldorf Düsseldorf Germany; ^3^ Institute for Biometrics and Epidemiology, German Diabetes Center, Leibniz Center for Diabetes Research at Heinrich Heine University Düsseldorf Düsseldorf Germany; ^4^ Austrian Heart Foundation Vienna Austria; ^5^ Ludwig Boltzmann Institute for Cardiovascular Research Vienna Austria; ^6^ German Center for Diabetes Research Partner Düsseldorf München‐Neuherberg Germany; ^7^ Centre for Health and Society, Faculty of Medicine Heinrich Heine University Düsseldorf Düsseldorf Germany

**Keywords:** HbA1c, meta‐regression analysis, precision medicine

## 1 INTRODUCTION

A growing body of evidence reveals opportunities as well as challenges for precision medicine in the prevention, diagnosis and treatment of diabetes mellitus.^1^, ^2^ Indeed, the heterogeneous and multifactorial nature of diabetes may favour a more patient‐centred approach. However, before additional significant efforts in precision diabetology are made, the potential of this approach with respect to precision treatment must be confirmed. In particular, it must be shown that the precision approach offers clinical benefits for the single individual with diabetes, when compared to standard care. Two conditions must be fulfilled for precision treatment to have a meaningful clinical effect^3^: First and foremost, heterogeneity in an individual's response to pharmacological treatment must be demonstrated. This is only possible with specialised trial designs (e.g., replicate crossover trials), which are not yet available in diabetology.^4^ As an alternative, results from parallel‐group placebo‐controlled trials can be used to evaluate the degree of treatment heterogeneity as measured by treatment response. Beyond variation within and among study participants, a larger variability in clinical outcomes would be expected in the active treatment groups compared to those allocated to placebo. Second, and if treatment heterogeneity exists, clinical predictors such as age, sex, or baseline HbA1c must be available to predict the best treatment for a single individual.

By applying these concepts, we have previously embarked on assessing the clinical benefit of current pharmacological treatment in type 2 diabetes with regard to improvement in glycaemic control, body weight reduction, and all‐cause mortality.^5^, ^6^, ^7^ The work presented here supplements those previous analyses by looking at the dose‐dependent variability of HbA1c. If heterogeneity in treatment response exists, then we would expect this heterogeneity to be larger with higher doses.

## 2 METHODS

### 2.1 Study selection and study outcome

We included randomised controlled trials (RCTs) which we recently analysed in two of our group's meta‐regression analyses.^5^, ^6^ As part of the inclusion criteria, we selected trials from previous systematic reviews that compared treatments for type 2 diabetes [alpha‐glucosidase inhibitors, dipeptidyl peptidase‐4 (DPP‐4) inhibitors, glucagon‐like peptide‐1 (GLP‐1) receptor agonists, metformin, sodium–glucose cotransporter‐2 (SGLT‐2) inhibitors, sulfonylureas, thiazolidinediones, combination therapies or others] to placebo, and reported on the variability of HbA1c.^8^, ^9^, ^10^, ^11^ In addition, studies needed to report data for a treatment applied at a minimum of two different doses and given for at least 24 weeks. Trials with multiple‐dose arms were included in order to investigate the relationship between dose and change in HbA1c. In trials with a single placebo arm but dose‐dependent information for multiple treatments, we pre‐selected one active treatment to avoid counting the placebo group more than once, which would artificially inflate the sample size. We excluded studies involving diabetic medications no longer used in clinical practice or which were withdrawn from the market, such as rosiglitazone or first‐generation sulfonylureas. Treatment arms with dose‐escalation schemes were assigned their respective maximal dose. Finally, we standardised dosage within trials from 0% (placebo arm dosage) to 100% (the maximal dose in the respective trial).

Our primary outcome of interest was the dose‐dependent variability of HbA1c, measured as Log[SD] after treatment in each trial arm. Additionally, the respective sample size and the mean HbA1c had to be available. Data extraction was carried out independently by two investigators, with any disagreements resolved by consensus with a third investigator.

### 2.2 Statistical analysis

To compare Log(SD)s across dosages, we applied the arm‐based model by Nakagawa et al.^12^ Weighted meta‐regression models were computed with a bias‐corrected outcome of (Log(SD) + 1/(2(*n* − 1))) with ‘*n*’ representing the sample size in each trial arm and ‘1/(2(*n* − 1))’, the bias correction. To assess increasing variability with dosage, the meta‐regression model used Log(SD) as the outcome and included percentage dose and Log(mean) of HbA1c as fixed effect covariates. The effect estimate of percentage dose is the primary parameter of interest; a value of 0 indicates constant variability of HbA1c across dosages and thus no potential for precision medicine. To allow for the correlations of dose‐dependent arms within a trial, a random intercept for the trial was included. Finally, we had to account for different sample sizes in the trial arms. For this task, each trial arm was weighted by its inverse‐variance weight of 2(*n* − 1). We used SAS, Version 9.4 (SAS Institute Inc., Cary, NC, USA) for all analyses.

## 3 RESULTS

After screening and removing duplicates from all RCTs considered in both previous meta‐regression analyses, we included 44 trials. These trials comprised 44 placebo and 101 different dosage arms. The 44 placebo arms included data from 6793 participants, while the 101 treatment arms provided data from 17,142 participants.

At baseline, and due to randomisation, the populations in the placebo and treatment arms were comparable with respect to mean age, proportion of male participants, mean body mass index (BMI) and mean known disease duration (Table 1). The most frequently used treatments were SGLT‐2 inhibitors (in 30 arms) and GLP‐1 receptor agonists (in 29 arms).

**TABLE 1:** Description of included trial arms, separated by placebo and treatment arms.

	Placebo (*N* = 44 arms)		Treatment (*N* = 101 arms)	
Variable	Number of missing arms	Median (Min/Q1/Q3/Max)	Number of missing arms	Median (Min/Q1/Q3/Max) or number (%)
Mean age at baseline (in years)	1	55.6 (52.0/54.3/58.6/63.2)	4	56.0 (50.6/54.6/58.5/63.2)
Proportion of male participants at baseline (in %)	1	54.0 (0.5/50.0/58.9/76.7)	4	55.9 (0.4/49.1/60.2/76.9)
Mean BMI at baseline (in kg/m^2^)	1	31.0 (24.9/28.7/32.7/34.7)	4	31.1 (24.4/29.2/32.3/35.0)
Mean known disease duration at baseline (in years)	10	6.6 (1.1/4.6/9.4/14.8)	22	6.6 (1.0/4.3/9.3/16.2)
Mean HbA_1c_ at baseline (in %)	0	8.2 (7.1/8.0/8.7/10.4)	0	8.1 (7.1/8.0/8.7/10.3)
Year	0	2013.5 (1998/2007/2016.5/2019)	0	2013 (1998/2006/2017/2019)
Duration of treatment (in weeks)	0	26 (16/24/28/206)	0	26 (16/24/26/206)
Treatment (drug class)				
Alpha‐glucosidase inhibitors	–	–	0	4 (4)
DPP‐4 inhibitors	–	–	0	10 (10)
GLP‐1 receptor agonists	–	–	0	29 (29)
Metformin	–	–	0	6 (6)
SGLT‐2 inhibitors	–	–	0	30 (30)
Thiazolidinediones	–	–	0	22 (22)
Number of treated individuals	0	110.5 (11/70/139/2266)	0	123 (11/80/163/2279)
Log(SD) of HbA_1c_ after treatment (in %)	0	0.11 (−0.98/−0.12/0.35/1.03)	0	0.02 (−0.82/−0.22/0.24/0.99)

Abbreviations: BMI, body mass index; DPP‐4, dipeptidyl peptidase‐4; GLP‐1, glucagon‐like peptide‐1; HbA1c, glycated haemoglobin A1c; SGLT‐2, sodium‐glucose co‐transporter 2.

**FIGURE 1:**
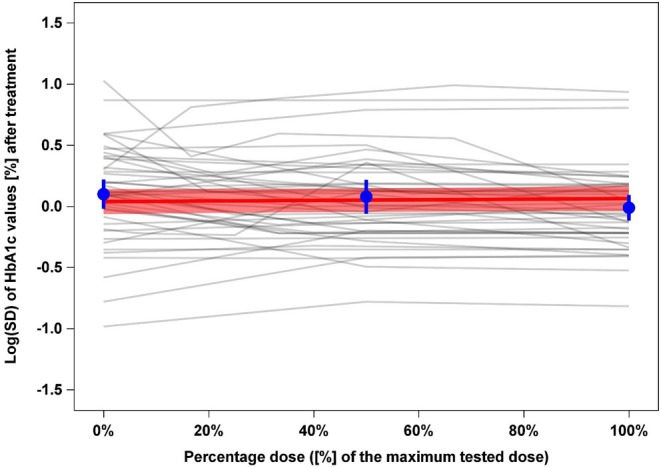
Line plot for the trajectories of raw Log(SD) values of HbA1c against percentage dose (0% = placebo arm, 100% = treatment arm with maximal dose). Each grey line demonstrates how the Log(SD) of HbA1c changes with different doses within a single trial. The red line is a fitted meta‐regression line representing the pooled estimate of the dose‐Log(SD) relationship across all trials, along with its pointwise 95% confidence interval (CI) (red shaded area). The blue points represent the average, unadjusted, unweighted mean Log(SD) at 0%, 50% and 100% dose levels, along with 95% CI. In summary, the figure shows how the variability of HbA1c changes with dose across all trials. Each grey line reflects this variability for every trial. The red line with its CI represent the average dose‐Log(SD) relationship estimated by the meta‐regression. Here, we observe a slight slope upwards, indicating a very small, non‐clinically relevant, increase in variability in HbA1c with higher doses.

Regarding the outcome of interest, the median Log(SD) of HbA1c across trials arms after treatment was 0.11% in the placebo arms and 0.02% in the treatment arms. This indicates a larger variability of HbA1c in the placebo arms. However, these analyses do not account for the fact that our data include trajectories of Log(SD) values with at least two treatment arms belonging to a single placebo arm. Figure 1 displays the respective trajectories of raw Log(SD) values of HbA1c against percentage dose, which show rather horizontal trends. Specifically, the effect estimate for percentage dose (representing the slope of the red line in the Figure) from the primary meta‐regression model is 0.025 [95% CI: −0.050; 0.099]. This finding suggests a very small increase across dosages.

## 4 INTERPRETATION AND CONCLUSION

This meta‐regression analysis, based on pooled data from 44 randomised trials including 23,935 participants, found no evidence of relevant dose‐dependent variability of HbA1c. Our unadjusted analysis, in fact, showed slightly higher variability in the 44 placebo groups when compared to the 101 treatment arms (0.11% vs. 0.02%, respectively). After fully adjusting for the actual treatment effect, different sample sizes, and the correlation of arms with trials, variability in the treatment arms was higher, though clinically irrelevant. This aligns with our previous investigations, which found that treatment heterogeneity (measured by clinical outcomes of dose‐*in*dependent HbA1c, body weight, and all‐cause mortality) was, at best, low.^5^, ^6^, ^7^ Considering the dose‐dependent variability of HbA1c, our study results demonstrate a limited potential for precision medicine with the current pharmacological treatment options for type 2 diabetes. However, these results should not be extrapolated to other clinically relevant endpoints such as quality of life or cardiovascular or renal outcomes.

There are some limitations to this work. First, our analysis used data from previous systematic reviews comparing glucose‐lowering therapy to placebo, and we did not update the search for more recent trials. However, these reviews thoroughly evaluated the current available evidence. Second, aggregated values from parallel‐group RCTs might not yield identical results to repeated crossover or N‐of‐1 trials, which use individual participant data. While these types of studies might be more suitable for identifying individual treatment effects and thus better for evaluating precision medicine, they are rarely performed; we are not aware of even a single trial in diabetology. Repeated crossover and N‐of‐1 trials may be particularly more challenging in diabetology due to the complex and progressive nature of diabetes, and the potential long‐term carryover effect observed in glucose‐lowering drugs such as GLP‐1 agonists. Because of the lack of dose‐dependent glycaemic treatment heterogeneity, we did not further explore the existence of clinical predictors (i.e., the second necessary condition for the clinical benefit of the precision treatment approach). In addition, a certain patient‐level heterogeneity (e.g., sex, age, baseline HbA1c) cannot be ruled out due to unavailability of patient‐level data, despite the low risk of bias in the randomisation process from the included trials. Future studies should challenge or corroborate our findings through individual patient‐data analyses.

Importantly, even though this work supports the notion of limited potential for the precision medicine approach (as shown by a minimal dose‐dependent variability in HbA1c), our results showing no treatment heterogeneity are not necessarily a drawback to available glucose‐lowering therapies. On the contrary, it reflects that, on average, most patients uniformly benefit from the current therapeutic options for treating type 2 diabetes.

## CONFLICT OF INTEREST STATEMENT

All authors declare no support from any industry, no financial relationships with any organisations and no other relationship or activity that could have influenced the present work. Maximilian Brockmeyer received congress travel expenses from Daiichi Sankyo and personal fees from Amgen, Sanofi‐Aventis and Novartis. Georg Wolff received congress travel expenses from Abbott. Oliver Kuss received honoraria for biostatistical education and consulting from Berlin‐Chemie.

## PEER REVIEW

The peer review history for this article is available at https://www.webofscience.com/api/gateway/wos/peer-review/10.1111/dom.70066.

## Data Availability

The data to reproduce all analyses are available from the authors on request.
